# RNAi-mediated CD40-CD154 interruption promotes tolerance in autoimmune arthritis

**DOI:** 10.1186/ar2914

**Published:** 2010-01-26

**Authors:** Xiufen Zheng, Motohiko Suzuki, Xusheng Zhang, Thomas E Ichim, Fei Zhu, Hong Ling, Aminah Shunnar, Michael H Wang, Bertha Garcia, Robert D Inman, Wei-Ping Min

**Affiliations:** 1 Departments of Surgery, Pathology, Microbiology and Immunology, University of Western Ontario, 1393 Western Road, London, Ontario, N6G 1G9, Canada; 2 Division of Rheumatology, Department of Medicine, Toronto Western Hospital, University Health Network, 1E423 - 399 Bathurst Street, Toronto, Ontario, M5T 2S8, Canada; 3 Medistem Inc, 9255 Towne Centre Drive, San Diego, CA 92121-3038, USA; 4 Multi-Organ Transplant Program, London Health Sciences Centre, 339 Windermere Road, London, Ontario, N6A, 5A5, Canada; 5 Transplantation and Regenerative Medicine, Lawson Health Research Institute, 339 Windermere Road, London, Ontario, N6A, 5A5, Canada

## Abstract

**Introduction:**

We have previously demonstrated that ex vivo inhibition of costimulatory molecules on antigen-pulsed dendritic cells (DCs) can be useful for induction of antigen-specific immune deviation and suppression of autoimmune arthritis in the collagen induced arthritis (CIA) model. The current study evaluated a practical method of immune modulation through temporary systemic inhibition of the costimulatory molecule CD40.

**Methods:**

Mice with collagen II (CII)-induced arthritis (CIA) were administered siRNA targeting the CD40 molecule. Therapeutic effects were evaluated by clinical symptoms, histopathology, Ag-specific T cell and B cell immune responses.

**Results:**

Systemic administration of CD40-targeting siRNA can inhibit antigen-specific T cell response to collagen II, as well as prevent pathogenesis of disease in both a pre- and post-immunization manner in the CIA model. Disease amelioration was associated with suppression of Th1 cytokines, attenuation of antibody production, and upregulation of T regulatory cells.

**Conclusions:**

These studies support the feasibility of transient gene silencing at a systemic level as a mechanism of *resetting *autoreactive immunity.

## Introduction

Rheumatoid arthritis (RA) is a chronic inflammatory and deforming joint disease that affects approximately 1% of adults. Although the precise etiology of RA has not been clearly ascertained, numerous studies support the concept that autoreactive T cells play a central role in the initiation and maintenance of the disease [1]. Advanced RA is treated with TNF-α inhibitors such as Infliximab or Embrel, however a significant proportion of patients do not respond [2]. These patients have shown some improvement following treatment with Abatacept, a clinically approved CTLA4-Ig, which is believed to inhibit antigen presenting cell (APC) co-stimulation of T cells by high affinity binding to CD80/86 [3]. Clinical responses induced by the co-stimulatory blockade support the rationale for targeting this pathway. In addition to the CD80/86-CD28 interaction, co-stimulation of T cell responses occurs through the CD40-CD154 interaction between APCs and T cells. CD40 signaling has been demonstrated to be critical in the initiation and progression of the rodent model of RA, collagen induced arthritis (CIA) [4]. It has been demonstrated that overexpression of CD154 (CD40L) on T cells correlates with higher disease activity [5], which is confirmed by studies showing treatment of mice with agonistic anti-CD40 Abs at the time of CIA induction exacerbates disease [6]. Conversely, administration of antagonistic anti-CD154 monoclonal antibody (mAb) prior to induction of CIA ameliorates the disease [7]. Suppression of the CD40-CD154 interaction has been shown to actually induce generation of T regulatory (Treg) cells [8]. Despite promising preclinical data, translation of CD40/154 blockade approaches has proved difficult due to the expression of CD154 on platelets, which causes risk of thromboembolic events. Accordingly novel methods of manipulating this interaction without evoking platelet reactions are needed.

Since the DC acts as the most potent APC, we have previously used siRNA to manipulate expression of immunological genes in antigen pulsed DCs to either upregulate or suppress immune responses in a specific manner [9,10]. However, ex vivo cellular manipulation is impractical for widespread use. In addition, numerous autoantigens are involved in clinical autoimmune diseases, thus adding another layer of complexity in terms of clinical development. Since CTLA4-Ig mediated co-stimulatory blockade induces remission of autoimmunity, we sought to determine whether a temporary suppression of CD40 expression by administration of siRNA may induce immune modulatory effects on RA that predispose towards reduction of immunity towards the autoantigen. Such an approach is based on the concept that a transient interruption of ongoing T cell activation during the initiation and progression of the autoimmune process may allow the host to default to a state of tolerance to the autoantigen.

In this study, we used a hydrodynamic protocol to systemically administer siRNA targeting CD40 in mice before and after administration of autoreactive antigen. We demonstrated antigen-specific immune modulation, as well as both inhibition of arthritic disease. These data support the possibility of temporary immune modulation in the context of autoimmunity.

## Materials and methods

### Animals

Male DBA/1 LacJ and BALB/c mice (The Jackson Laboratories, Bar Harbor, ME, USA), five weeks of age, were kept in filter-top cages at the Animal Care and Veterinary Services Facility at the University of Western Ontario, according to the Canadian Council for Animal Care Guidelines. Mice were fed food and water *ad libitum *and allowed to settle for two weeks before initiation of experimentation, which had ethical approval from the university review board.

### CIA Model

DBA/1 LacJ mice, seven weeks of age, were intradermally immunized (Day 0) at the base of the tail with 200 μg of bovine type II collagen (CII) (Sigma-Aldrich, St. Louis, MO, USA) with complete Freund's adjuvant (CFA) (Sigma). On Day 21 after priming, the mice received an intraperitoneal booster injection with 200 μg. Mice were examined visually three times per week for the appearance of arthritis in the peripheral joints, and an arthritis score index for disease severity was given as follows: 0 - no evidence of erythema and swelling; 1 - erythema and mild swelling confined to the mid-foot (tarsals) or ankle joint; 2 - erythema and mild swelling extending from the ankle to the mid-foot; 3 - erythema and moderate swelling extending from the ankle to the metatarsal joints; 4 - erythema and severe swelling encompassing the ankle, foot, and digits. The maximum possible score per mouse was 16. The severity of the arthritis was also determined by the quantification of the paw swelling measured with a dial gauge caliper. Scoring was done by two independent observers, without knowledge of the experimental and control groups.

### DC Cultures

DCs were generated from bone marrow progenitor cells as previously described [11]. Briefly, DCs were cultured from bone marrow cells in six-well plates (Corning Inc., Corning, NY, USA) at 4 × 10^6 ^cells/well in 4 mL of RPMI 1640 medium supplemented with 2 mM L-glutamine, 100 U/ml penicillin, 100 μg of streptomycin, 50 μM 2-ME, and 10% Fetal calf serum (FCS) (all from Invitrogen, Mississauga, Ontario, Canada). Recombinant granulocyte macrophage clony-stimulating factor (GM-CSF) (10 ng/mL; PeproTech, Rocky Hill, NJ, USA) and recombinant mouse interleukin 4 (IL-4) (10 ng/mL; PeproTech) were added to the culture medium as growth factors. All cultures were incubated at 37°C in 5% humidified CO_2_. Non-adherent cells were removed after 48 h of culture (Day 2) and fresh medium was added every 48 h.

### siRNA design and gene transfection

siRNA sequence (TGTTCCACTGGGCTGAGAA), specifically targeting the CD40 gene, was designed and synthesized by Dhmarcon (Dharmacon, Inc., Chicago, IL, USA). CD40 siRNA-expressing vectors were generated using the Silencer Express Kit (Ambion Inc, Austin, TX, USA).

DCs (10^6^cells/well) were plated in a 12-well plate one day before transfection. On Day 6, 3 μl of 20 μM annealed CD40 siRNA and 5 μL of transfect reagent Genesilener (Gene Therapy Systems, San Diego, CA, USA) were separately diluted with 50 μL serum-free medium RPMI 1640, and then mixed rapidly and incubated at room temperature for five minutes to form a complex. The above siRNA-Genesilencer complex was added to the DCs. GL2 siRNA was used as a negative control. After four hours incubation, an equal volume of RPMI 1640 (500 μl) supplemented with 20% FCS, 20 ng/mL GM-CSF and 20 ng/mL IL-4 was added to the cells. Twenty-four hours after transfection, cells were collected to determine gene expression in DCs.

### Administration of siRNA

DBA/1LacJ mice were treated with CD40 siRNA or control siRNA on Day -2 or Day 2 using a hydrodynamic method [12]. In brief, 50 μg siRNA was diluted in 1 ml Phosphate buffered saline (PBS) and quickly injected into mice via tail veil in 10 s.

### Mixed Leukocyte Reaction (MLR)

At Day 6 of culture, bone marrow-derived DCs from DBA/1 LacJ mice were transfected with CD40 siRNA or control siRNA. Transfected DCs were irradiated (3,000 rad) and seeded in triplicate in a flat-bottom 96-well plate (Corning) for use as stimulator cells. Spleen T cells from BALB/c mice were isolated by gradient centrifugation over Ficoll-Paque (Amersham Pharmacia Biotech, Montreal, Quebec, Canada) and added as responders (2 × 10^5 ^cells/well). The mixed lymphocytes were cultured at 37°C for 72 h in 200 μL of RPMI 1640 supplemented with 10% FCS, 100 U/ml of penicillin, and 100 μg/mL of streptomycin and pulsed with 1 μCi/well of ^3^H-labelled thymidine (Amersham Pharmacia Biotech) for the last 18 h of culture. Finally, cells were harvested onto glass fiber filters (Perkin Elmer Life Science, Turku, Finland), and the radioactivity incorporated was measured using a Wallac Betaplate liquid scintillation counter (Beckman, Fullerton, CA, USA). Results were expressed as mean counts per minute of triplicate cultures ± SEM.

### CII-specific T cell response

T cell responses to CII in subsequent groups of mice were measured by ^3^H -thymidine incorporation in the presence of CII. T cells were isolated from spleen and lymph node by gradient centrifugation over Ficoll-Paque (Amersham Pharmacia Biotech). Unfractionated lymph node cells were cultured in 96-well plates at a concentration of 2 × 10^5 ^cells/well for 72 h in the presence or absence of CII antigen. Cells were cultured for two days, and then cells were pulsed with 1 μCi of ^3^H-thymidine (Amersham Pharmacia Biotech) for another 18 h of culture. Cells were harvested onto glass fiber filters, and incorporated radioactivity was quantified using a Wallac Betaplate liquid scintillation counter.

### Anti-CII antibody measurement

CII-specific Abs were detected using a standard indirect ELISA in which 500 ng of CII was absorbed to each well of a 96-well microtitre plate. Serial dilutions of immune mouse serum were added to the appropriate wells in duplicates and incubated overnight at 4°C. Dilutions of serum were 1:100 to 1:10,000. To develop the ELISA, horseradish peroxidase-conjugated goat anti-mouse IgG Fc and ortho-phenylenediamine dihydrochloride substrate buffer (Sigma) were used. An OD into each well was measured at 450 nm wavelength in an ELISA plate reader (Bio-Rad, Hercules, CA, USA).

### Cytokine quantification

CD40 siRNA- or control siRNA-transfected DCs derived from DBA/1 LacJ mice were cultured with the allogeneic (BALB/c) T cells or alone for 48 h. The supernatants were collected and examined for cytokines IL-2, IFN-γ and IL-4) by ELISA. Cytokine-specific ELISA (Endogen, Rockford, IL, USA) was used for detecting cytokine concentrations in culture supernatants according to the manufacturer's instructions using a Benchmark Microplate Reader (Bio-Rad Laboratories Ltd., Mississauga, ON, Canada).

### Histology

Paws from experimental and control groups of freshly dissected mice were removed and joint tissues were immersion-fixed in buffered formalin in 0.15 M PBS (pH 7.4) and decalcified with 5.5% EDTA. The specimens were processed for paraffin embedding in paraplast (BDH, Poole, Dorset, UK) as routine procedure. Serial paraffin sections throughout the joint were cut at 5 μm thickness on a microtome. Sections were stained with haematoxylin and eosin (H&E) for assessment of histological damage. The severity of joint damage was scored in a blinded manner as follows: 0 = normal, 1 = moderate infiltration with discrete erosions, 2 = severe infiltration and moderate erosions with preserved joints structure, and 3 = severe erosions with loss of joint architecture.

### Flow cytometry

Phenotypic analysis of DC was performed using flow cytometry on a FACScan (Becton Dickinson, San Jose, CA, USA). The cells were stained with FITC-, PE- or PE-cy5-conjugated mAbs against surface markers associated with DC maturation, these include: anti-mouse CD11c, anti-mouse CD40, anti-mouse CD80 (Cedarlane Laboratories, Mississauga, ON, Canada). Tregs were analyzed by triple staining with mAb against with Foxp3-FITC, CD25-PE, and CD4-CY5, followed by flow cytometry analysis. Ig of the same isotype was used as a control.

### Statistical analysis

Data are expressed as mean ± SEM. Differences between different groups of mice were compared using the Student t-test for gene expression, antibody, cytokine and histological scores or the Mann-Whitney U test for nonparametric data. A *P-*value less than 0.05 was considered significant.

## Results

### Knockdown of CD40 reduces allogenicity in DCs

**Figure 1:**
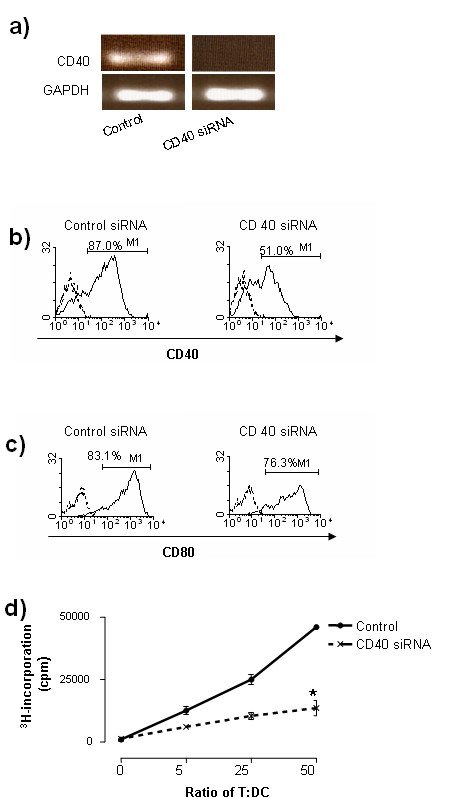
**Knockdown of CD40 reduces allogenicity in DCs**. DBA-derived DCs were cultured as described in Materials and methods. DCs were transfected with CD40 siRNA using Genesilencer. Forty-eight hours after gene silencing, reduced gene expression of CD40 was detected by RT-PCR **a) **and flow cytometry **b)**, respectively, without affecting CD80 gene expression determined by flow cytometry **c)**. CD40-silenced DCs were also used to co-culture allogeneic (BALB/c) T cells. T cell proliferation was assessed in MLR **d)**. Data are presented as mean ± SEM. Results represent one of three experiments. * = *P *< 0.05 versus control siRNA.

Immature DCs are characterized by relatively low expression of stimulatory signals and higher levels of inhibitory signals to T cells [13]. Previously we demonstrated feasibility of manipulating such signals using siRNA on DCs in order to generate immunomodulatory DCs in a reproducible manner by silencing IL-12p35 [14]. We have postulated that specific gene knock-down may be a more attractive and defined means of immune modulation as opposed to chemically generated immature DCs, which we have also demonstrated to inhibit disease progression in CIA [15]. Here we sought to develop inhibitory DCs through gene silencing of CD40. DCs were generated from bone marrow progenitors by standard IL-4 and GM-CSF culture and transfected on Day 6 with CD40-siRNA. Specific knockdown of CD40 mRNA and protein was seen in DCs by RT-PCR (Figure 1A), and flow cytometry (Figure 1B), respectively, as compared to DCs transfected with control scrambled siRNA. Silencing was specific in that CD 40 siRNA did not change gene expression of CD80 in DCs (Figure 1C).

Stimulation of T cell responses by DCs requires TCR engaging molecules (MHC) and co-stimulatory molecules CD40 [16]. CD40 is a critical co-stimulatory molecule, thus knockdown of this molecule may impair DC's ability to activate T cell response. To test this hypothesis, we used CD40-silenced DCs as allostimulators in MLR. As seen in Figure 1D, CD40-silenced DCs exhibited markedly inferior allostimulatory activity as compared to control DCs.

### In vivo immunomodulation by administration of CD40 siRNA

**Figure 2:**
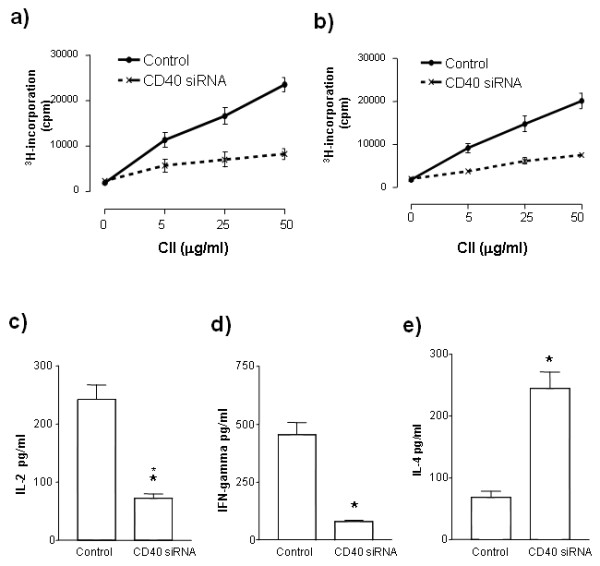
***In vivo *immunomodulation by administration of CD40 siRNA**. **a) **and **b)**: CII-specific T cell responses by CD40 siRNA. DBA mice (seven mice per group) were injected with 50 μg of CD40 siRNA or control siRNA using hydrodynamic injection method. Two days after siRNA treatment, mice were immunized with 200 μg CII antigen. T cells from lymph nodes (A) and spleens (B) were isolated from the mice treated with CD40 siRNA or control siRNA. The antigen-specific T cells responses were assessed in the presence of CII. T cell proliferation was determined by 3H-incorporation assay, as described in the Materials and Methods. **c)**, **d) **and **e)**: The differentiation by CD40 siRNA. The supernatants from above cultures were harvested. The cytokines of IL-2 (C), INF-γ (D) and IL-4 (E) produced by T cells in the CII-specific response were detected by ELISA, as described in Materials and methods. Data are presented as mean ± SEM. Results represent one of three experiments. * = *P *< 0.05 versus control siRNA.

Our previous findings in other systems suggest that manipulation of DC co-stimulatory molecules, either with chemicals [15] or siRNA [14,17] provides a simple and reproducible method of *in vivo *inducing antigen-specific immune deviation. We sought to test the feasibility of immunomodulation with CD40 knockdown in the CIA model. However, unlike our previous experiments in which CD40 knockdown was performed *ex vivo *on antigen-pulsed DC to induce immune deviation [10], here siRNA to CD40 was systemically administered two days prior to immunization with CII. Using the hydrodynamic injection method, 50 μg of siRNA was administered via the tail vein two days before immunization. Proliferative recall response to CII was performed to assess modulation of T cell activation under the cover of CD40 silencing. T cells isolated from lymph nodes (Figures 2A) and spleens (Figures 2B) exhibited reduced proliferation from animals pretreated with CD40 siRNA but not controls. In contrast, no modulation of response to T cell activation with anti-CD3/CD28 in vitro was observed, indicating that immune modulation was not associated with unspecific T cell suppression (data not shown).

Blockage of the CD40-CD154 interaction has been reported to alter cytokine production [16]. We sought to assess whether *flooding *the mice with CD40 siRNA would affect cytokine differentiation. Treatment with CD40 siRNA remarkably inhibited Th1 recall response, as evidenced by decreased IL-2 (Figure 2C), and IFN-γ (Figure 2D). The Th2 cytokine IL-4 was increased in the mice treated with CD40 siRNA (Figure 2E). These data imply that siRNA treatment induces immune modulation *in vivo *including suppression of CII-Ag-specific T cell responses as well as suppression of Th1 differentiation.

### Prevention of autoimmune arthritis by treatment with CD40 siRNA

**Figure 3:**
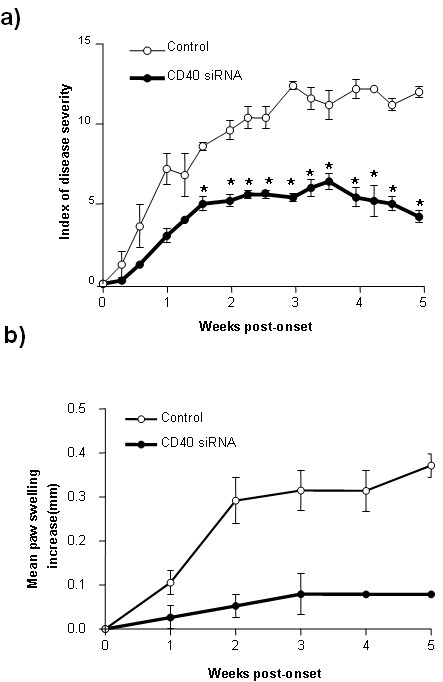
**Preventing autoimmune arthritis by CD40 siRNA**. DBA mice (seven mice per group) were injected with 50 μg of CD40 siRNA or control siRNA using the hydrodynamic injection method. Two days after siRNA treatment, mice were immunized with 200 μg CII antigen, two times on Day 0 and Day 21, respectively. Treatment of CD 40 siRNA was repeated on Day 7 after first CII immunization. Disease severity was scored from the day of last immunization to five weeks **a)**. The swelling of joints of CIA mice was determined by the thickness of each hind paw measured with caliper **b)**. Data are presented as mean ± SEM. Results represent one of three experiments. * = *P *< 0.05 versus control siRNA.

In previous studies we demonstrated that chemical manipulation in DCs through inhibition of IKK, which is responsible for CD40 upregulation in DC, can result in generation of antigen-specific immune regulation and suppression of pathology in the CIA model [15]. Here we tested whether the systemic administration of CD40 siRNA may have a similar effect. DBA mice were treated with a dose of 50 μg CD40 siRNA two days before immunization with CII antigen and subsequently CD40 siRNA was administered on Day 7 after immunization with CII. After a boost immunization on Day 21, onset of CIA was assessed. The disease onset occurred around Day 28 in control siRNA treated group, versus Day 35 in CD40 siRNA treated group, as judged by erythema and swelling of joints. Inhibition of arthritis clinical score was observed in the mice treated with CD40 siRNA, as opposed to control siRNA-treated mice which exhibited no inhibition (Figure 3A). Additionally, the degree of joint swelling was remarkably attenuated (Figure 3B) in the mice treated with CD40-siRNA. These data suggest that systemic administration of CD40 siRNA may prevent autoimmune arthritis.

### Intervention of autoimmune arthritis by treatment with CD40 siRNA

**Figure 4:**
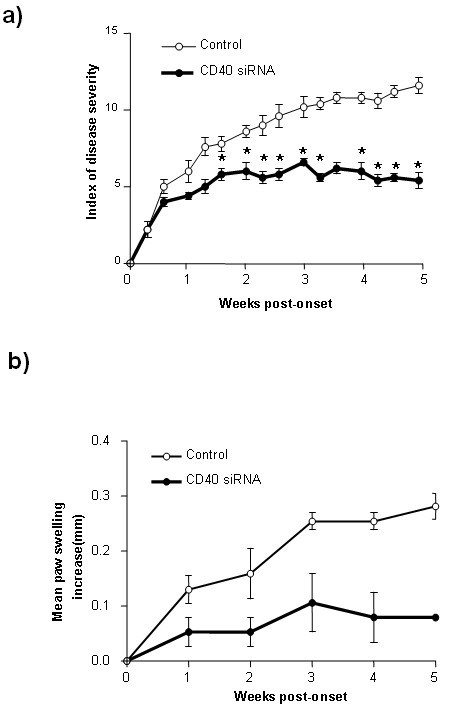
**Attenuated disease development by CD40 siRNA**. DBA mice were immunized with 200 μg CII antigen, two times on Day 0 and Day 21, respectively. Two days after CII immunization, the mice were treated with 50 μg of CD40 siRNA using the hydrodynamic injection method. siRNA treatment was repeated two weeks later. Disease severity was scored from the day of last immunization to five weeks **a)**. The swelling of joints of CIA mice was determined by the thickness of each hind paw measured with caliper **b)**. Data are presented as mean ± SEM. Results represent one of three experiments. * = *P *< 0.05 versus control siRNA.

In order to explore the protective effect of CD40 siRNA for potential clinical use, we treated mice at the stage of post-initiation of the autoimmunity. Experimentally, DBA mice were treated with CD40 siRNA two days after CII immunization and repeated administration of siRNA two weeks after first treatment. The average day of disease onset was 31 days in the mice treated with CD40 siRNA. After treatment with CD40 siRNA, the disease score was significantly reduced as compared with mice treated with control siRNA (Figure 4A). Furthermore, the joints were less severe with decreased swelling of the joints (Figure 4B). These data suggested that siRNA may possess protective effects in a post-immunization stage of autoimmune arthritis.

### Histological assessment

**Figure 5:**
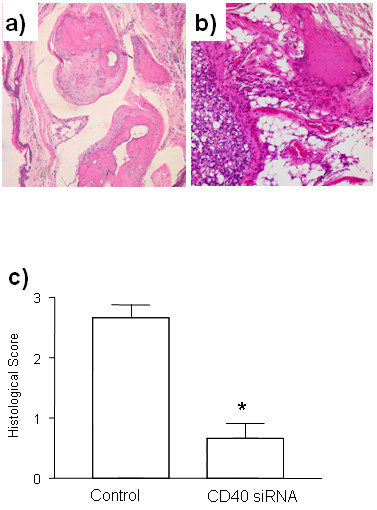
**Histological changes in CIA mice after treatment with CD40 siRNA**. At the end point of observation, mice were sacrificed. The joints from the mice treated with CD40 siRNA **a) **and control siRNA **b) **were collected, fixed, and stained with H&E. The hisopathological change was scored **c)**. Original magnification × 100. Results are representative of seven mice.

To confirm the therapeutic effects of CD40 siRNA, we next examined pathological changes in joints of CIA mice. We observed that control mice displayed severe bone erosion, pannus formation, and synovitis (Figure 5A). Furthermore, in control mice a marked neutrophilic and mononuclear cell infiltration was seen. In contrast, joints in mice treated with CD40 siRNA revealed, in most cases, markedly attenuated morphological changes and cellular infiltration, and the preservation of normal-appearing cartilage (Figure 5B). The average histological score was much lower in the group treated with CD40 siRNA as compared to the control siRNA group (Figure 5C).

### Reduced CII antibody production and generation of Treg by treatment of CD40 siRNA

**Figure 6:**
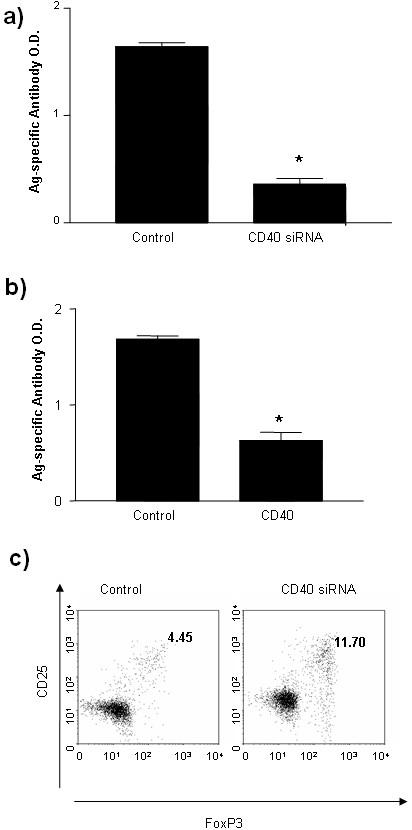
***In vivo *immune modulation by CD40 siRNA**. **a) **and **b) **Inhibition of CII-specific antibody production in arthritis mice following CD40 siRNA. DBA mice were immunized with CII antigen and treated with CD40 siRNA or control siRNA, as described in Figure 3 or Figure 4. Sera were collected at the end point of experiments (five weeks after second immunization). The CII-specific antibodies from the mice in Figure 3 (A) and in Figure 4 (B) were detected by ELISA, as described in Materials and methods. **c)**. Generation of Treg in arthritis mice following CD40 siRNA. DBA mice were immunized with CII antigen and treated with CD40 siRNA or control siRNA, as described in Figure 3. T cells were harvested from lymph nodes in the mice at the end point of experiments (five weeks after the second immunization). T cells were stained with mAbs against CD4, CD25 and Foxp3, respectively, and analyzed by flow cytometry. Results represent one of three experiments.

In order to dissect the mechanisms underling the prevention and treatment of autoimmune arthritis by CD40 siRNA, we analyzed antibody levels in the CIA mice treated with CD40 siRNA. The reduction of CII antibody production was observed in the groups treated with CD40 siRNA either before immunization with CII (Figure 6A) or post immunization with CII (Figure 6B).

Furthermore, CD40 siRNA can inhibit immune response *in vitro *(Figure 1) and *in vivo *(Figure 2), which could be induced by an active immune suppression through Treg cells. To test this hypothesis, we assessed Treg cells in the CIA mice that were treated with CD40 siRNA or control siRNA. Treatment with CD40 siRNA enhanced CD4^+^CD25^+^Foxp3^+ ^Treg cells generation (Figure 6C). These data imply that Treg may contribute to immune modulatory effects of CD40 siRNA therapy.

## Discussion

This report is the first to our knowledge to describe systemic inhibition of CD40 by siRNA as a means of interrupting autoimmune processes in order to up-regulate the natural tendency of the immune system to re-equilibrate tolerogenic mechanisms. We observed that administration of siRNA targeting CD40 could induce *in vivo *inhibition of proliferative and cytokine recall response to the joint antigen CII, as well as prophylactically inhibit disease. Mechanistically it appeared that induction of Treg cells was associated with suppression of autoimmunity.

The CD40-CD154 interaction is involved in the initiation of CIA. The major approach to intervening in this interaction has been use of CD154-specific blocking mAbs, which prevent T cell priming. This therapy is highly effective in mouse models [4,18], although the overall benefit of interruption of CD40-CD154 association is reduced when Abs is administrated after disease establishment. However, anti-CD154 treatment prevents relapses of ongoing disease [18]. Despite the great promise displayed for CD154 Abs in a mouse CIA model, a particular challenge in clinical use has been thromboembolism [4,18]. Another approach has been the use of blocking peptides. Unfortunately, this approach was restricted by the high concentrations needed for in vivo effects [19]. Using mAbs against CD40 such as the anti-human CD40 antagonist mAb, ch5D12, showed promise in autoimmune disease. New intervention strategies that spare normal immune function while blocking damaging effects of CD40 signaling are desirable. RNA inference may provide a novel approach to blocking the CD40 signal.

In the area of tolerance induction, numerous approaches have been attempted, however, to our knowledge, none have passed registration trials. Strictly antigen-specific approaches are deficient because in many situations numerous autoantigens contribute to disease. In the context of RA, while CII is sufficient to induce an RA-like disease in animal models, multiple proteins have been implicated in the human RA [1]. Examples of autoantigens in human RA include citrullinated fibrinogen, hsp47 and 60 [20,21]. The second problem is that induction of Ag-specific tolerance can only be achieved by limited means even if antigens are known; for example, oral tolerance [22], intravenous tolerance, or as we previously published, *ex vivo *pulsing of DCs with autoantigen. These methods are either not practical or weakly effective.

The method used in the current study is based on our previous observations that manipulation of CD40 on DC can be tolerogenic [10,23]. Accordingly, we sought to determine if systemic inhibition through flooding the body with siRNA can achieve a therapeutic effect. This method has several drawbacks: First, hydrodynamic administration is clinically impossible. Second, while we demonstrated inhibition of disease, complete tolerance was not achieved. Third, the possibility of non-specific immune suppression during administration of CD40 siRNA is a possibility. There are ways to address these concerns which will be studied in subsequent experiments. We previously reported successful administration of nanoparticles containing siRNA to DCs by the use of DEC-205 antibody coated immunoliposomes [23]. By selectively delivering the immune modulatory siRNA to the targeted DCs, it may be feasible to circumvent the clinical barriers associated with hydrodynamic administration. While complete inhibition of disease was not observed, the finding that administration of siRNA even after disease initiation occurred was effective suggests that the temporary CD40 inhibition may have potent therapeutic effects. While it is tempting to suggest multiple administrations, this would risk the possibility of compromising immunity towards pathogenic agents, as seen with the TNF-inhibitors causing reappearance of infectious diseases such as tuberculosis. One method of avoiding the need for multiple immune suppressions may involve co-immunization of a recipient with autoantigens under the cover of CD40 siRNA. This may be one method of *recycling *previously tried antigen-specific agents that demonstrated some efficacy in clinical trials but were not sufficient to warrant marketing approval.

It is recognized that CD40 is expressed by B cells, macrophages, and some T cells. The effects seen after inhibition of CD40 might be attributable to a direct effect on CD40 in B cells or an indirect effect through lower activation of T cells. We consider that one attractive possible mechanism by which systemic CD40 inhibition mediates immune modulation is through the up-regulation of Treg cells. We have demonstrated increased numbers of Treg cells in CD40 siRNA treated mice. It is conceptually appealing to ask whether these Treg cells arise due to the presence of autoantigen in the absence of co-stimulatory molecules. Indeed, DCs in RelB knockout mice, which lack CD40, have been demonstrated to induce infectious tolerance through induction of Treg cells [24].

## Conclusions

In conclusion, we have demonstrated a simple and easy to induce a method of selectively inhibiting disease progression in the CIA model of RA through systemic administration of CD40 siRNA. The possibility of using such *semiselective *methods of immune modulation which do not require long-term administration may be a more natural and easily tolerated means of treating autoimmune disease than current techniques in which immune modulators are given on a continuous basis.

## Abbreviations

APC: antigen presenting cell; CFA: complete Freund's adjuvant; CII: type II collagen; CIA: collagen-induced arthritis; DCs: dendritic cells; FCS: Fetal calf serum; GM-CSF: Granulocyte macrophage colony-stimulating factor; H&E: haematoxylin and eosin; IL-4: interleukin 4; mAb: monoclonal antibody; MHC: major histocompatibility complex; MLR: Mixed Leukocyte Reaction; PBS: Phosphate buffered saline; RA: Rheumatoid arthritis; siRNA: small interfering RNA; Treg: regulatory T cell.

## Competing interests

The authors declare that they have no competing interests.

## Authors' contributions

XiZ, MS, XuZ, FZ, HL, AS and MHW performed research. XiZ, TEM, RDI and WM analyzed data and wrote the paper; WM, RDI and XiZ designed the research.
